# Effect of adding abdominal bracing to spinal stabilization exercise on lumbar lordosis angle, extensor strength, pain, and function in patients with non-specific chronic low back pain: A prospective randomized pilot study

**DOI:** 10.1097/MD.0000000000035476

**Published:** 2023-10-13

**Authors:** Han Soo Park, Si Won Park, Jae-Keun Oh

**Affiliations:** a Sports Medicine Laboratory, Korea National Sports University, Yangjae-daero, Songpa-gu, Seoul, Republic of Korea.

## Abstract

**Trial design:**

This study investigated the effect of adding abdominal bracing to spinal stability exercise in patients with chronic low back pain (CLBP). This prospective, randomized pilot study included 67 patients and was conducted at the sports medicine center of a single hospital.

**Methods:**

The abdominal bracing group (ABBG) underwent spinal stability exercise with abdominal bracing (N = 33), comprising 50 minutes training twice a week for 24 weeks. The control group performed only spinal stability exercise (N = 34) for 50 minutes twice a week for 24 weeks. The ABBG received abdominal bracing training at each session and applied abdominal bracing during the spinal stability exercise. The lumbar lordosis angle (LLA) and spine extensor muscle strength were measured. Spinal flexion angles were measured every 12° from 0° to 72°. The visual analog scale score and Oswestry disability index were measured before treatment and at 12 and 24 weeks after treatment.

**Results:**

The LLA increased over time in both the groups but was not significantly different between the groups. Spine extensor strength was improved over time in both the groups, and an interactive effect was observed at a spinal flexion angle of 60° and 72°. Pain and function were also improved over time in both the groups, but the effect was stronger in the ABBG than in the control group. In patients with CLBP, spinal stability exercise changed the LLA.

**Conclusions:**

Although adding abdominal bracing to spinal stability exercise did not affect the changes in the LLA, abdominal bracing improved the spinal extensor strength, pain, and function in patients with CLBP. Therefore, it is recommended to add abdominal bracing to spinal stability exercise to maintain the lordosis angle and to improve CLBP symptoms.

## 1. Introduction

Low back pain (LBP) is prevalent in modern society; back pain not only causes disability in daily life but also degrades the quality of life and can result in psychological problems, including depression and anxiety.^[1,2]^ Chronic low back pain (CLBP) is generally defined as pain that persists beyond the normal healing period of more than 3 to 6 months.^[3]^ According to a 2017 survey, the global prevalence of LBP was 7.5%, and the years lived with a disability was 42.5 million, peaking at the age of 45 to 49 years and declining thereafter.^[4]^

Although various causes of CLBP have been reported, muscle weakness and imbalance around the lumbar spine and changes in the lumbar lordosis angle (LLA) are the main causes of CLBP.^[5–8]^ Weakened spinal extensor muscle strength also acts as a risk factor for LBP.^[9,10]^ In clinical practice, nonsteroidal anti-inflammatory drugs, opioids, neurotropic medications, steroid injections, manipulation, and mobilization have been employed to relieve the symptoms of CLBP.^[11–13]^ However, it is important to determine the root cause of CLBP recurrence.

It is known that exercise intervention not only improves muscle strength around the lumbar spine but also positively changes the LLA.^[6,14,15]^ According to a recent literature review, exercise intervention was more effective than other treatments in treating symptoms of CLBP. Moreover, among exercise interventions, spine stabilization exercise (SSE) was more effective than cardiorespiratory and combined exercise programs.^[16]^ Lack of lumbar stability reportedly causes chronic and recurrent LBP, and lumbar SSE is an effective method for maintaining lumbar spine stability.^[17,18]^ Furthermore, SSE is a suitable method for patients with CLBP to reduce pain and improve the ability to perform activities of daily living, as it can safely and effectively improve neuromuscular control, muscle strength, and muscular endurance of the lumbar stabilization muscles and can be personalized according to individual patient characteristics.^[19–21]^

In addition to SSE, abdominal bracing (ABB) is effective in improving lumbar stability.^[22]^ ABB increases spinal stabilization by activating the deep abdominal muscles, including the internal oblique (IO) and transverse abdominis (TrA) muscles, and increasing intra-abdominal pressure (IAP).^[23–25]^ Applying SSE and ABB to patients with CLBP has promoted symptom improvement and made exercise tolerable, but few studies have shown the effect of ABB when applied to patients with CLBP performing SSE.^[26]^ Therefore, this study aimed to investigate the effects of adding ABB to SSE on the LLA, lumbar extension strength, pain, and function in patients with CLBP after 24 weeks. We hypothesized that the group that performed both SSE and ABB would show better improvements than the group that performed SSE without ABB.

## 2. Methods

In this prospective randomized pilot study, randomization was performed by a therapist who was not involved in the study at the time of the first participant interviews. After baseline measurements, randomization was conducted by distributing blank envelopes with 2 group numbers to the participants, categorized by sex, and subsequently randomly assigning patients to either the abdominal bracing group (ABBG, performed SSE with ABB) or control group (CG, only performed SSE). The blinding method could not be applied to the researcher (S.W.) who conducted the intervention. However, all other researchers were blinded to group allocation and statistical analyses.

**Figure 1.**
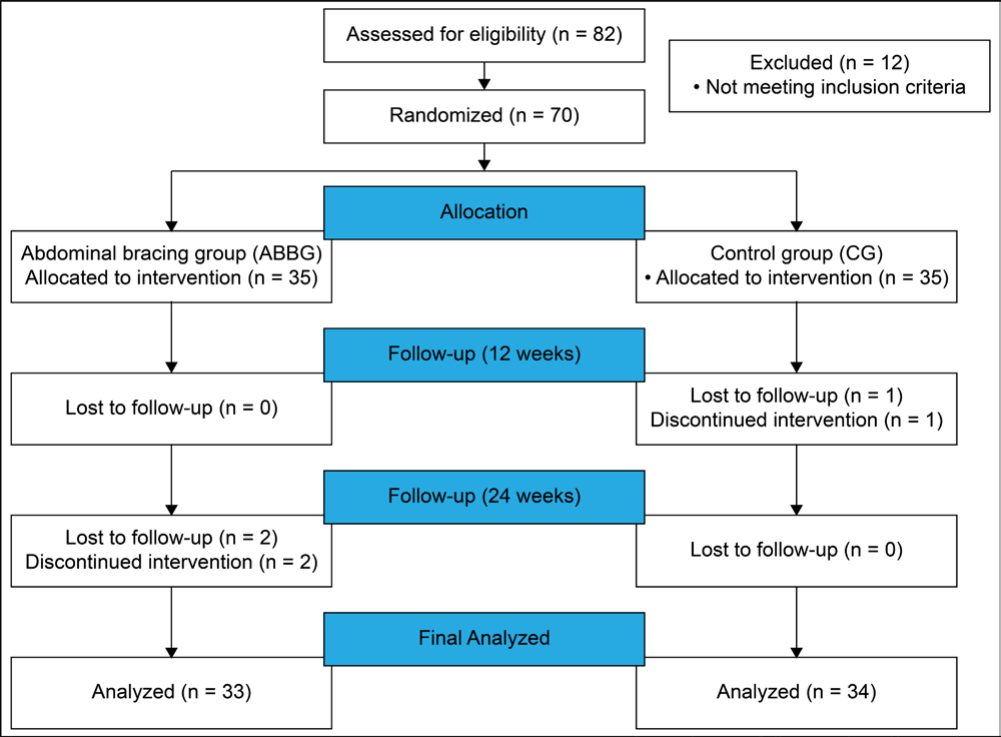
Flow diagram showing the progress of the study.

Before participating, the purpose, methods, and risks of the study were explained to the participants, and written informed consent was obtained. The study procedure was approved by the Institutional Review Board of the Nanoori Hospital (Gangnam-gu, Seoul, Republic of Korea) (NR-IRB 2018-016) and conforms to the guidelines of the Declaration of Helsinki. The trial was registered at the Clinical Research Information Service under code KCT0007777 on 06/10/2022. A CONSORT diagram for this study is shown in Figure 1.

### 2.1. Participants

In this study, the sample size was obtained using G* power version 3.1 (University of Dusseldorf, Dusseldorf, Germany), with an effect size (ES) of 0.40, a power of 0.95, and a significance level of.05. Seventy participants were selected considering a drop rate of 30%. Participants diagnosed with CLBP at an orthopedic or neurosurgery outpatient clinic were recruited.

The inclusion criteria were participants aged ≥20 years who complained of CLBP for 3 months or longer. The exclusion criteria were an age of ≥65 years, back pain accompanied by neurological symptoms, history of back surgery, visual analog scale score <4, severe scoliosis with Cobb angle exceeding 10º, severe arthritis of the lower extremities (hip, knee, and ankle), and pregnancy.

### 2.2. Intervention

**Figure 2.**
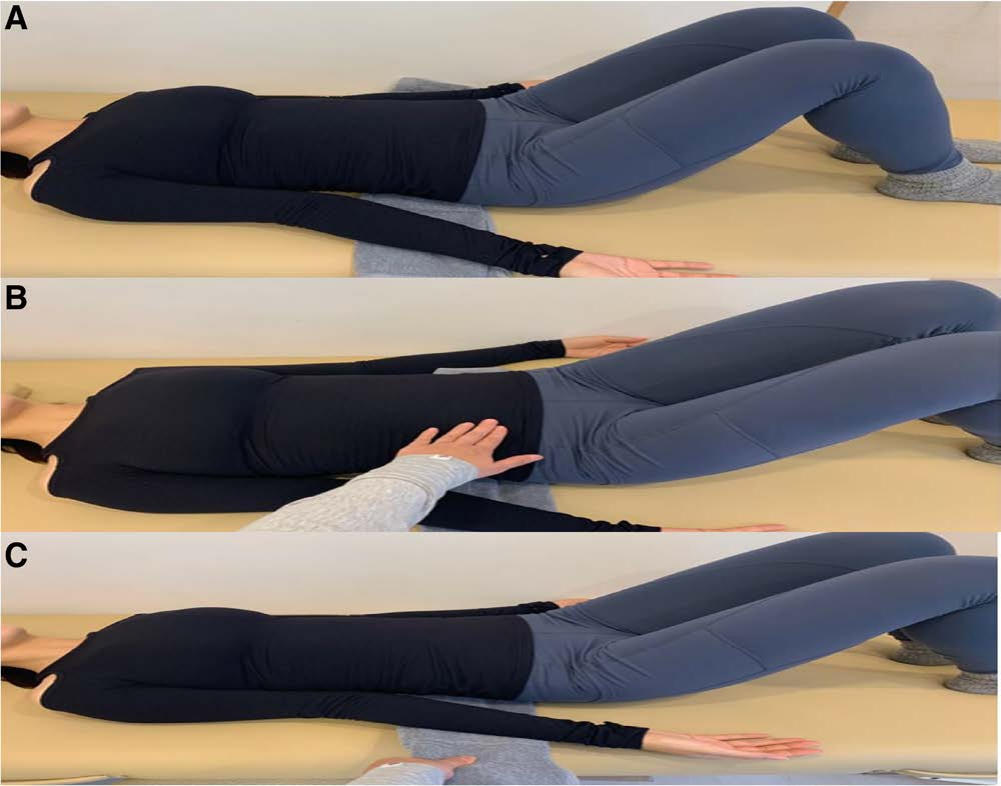
Abdominal bracing training comprised three stages: (A) Preparation phase: a towel is placed underneath the participant torso while in the supine position with their knees bent. (B) Relaxation phase: after the therapist puts his hand on the participant abdomen, the participant pushes his hand while keeping the lower back free from the floor and expanding the rib cage and abdomen as much as possible. (C) Contraction phase: when the therapist pulls the towel, the participant maintains abdominal pressure to prevent the towel from coming out as much as possible. This is repeated 10 times for 10 s each time.

Both the groups visited a sports medical center (Nanoori Sports Medical Center, Gangnam-gu, Seoul, Korea) and participated in an SSE program comprising exercise for 50 minutes twice a week for 24 weeks. In each session, the ABBG also underwent ABB, which comprised preparation, relaxation, and contraction phases, before performing the SSE program (Figs. 2A–C). In the ABBG, ABB was applied to the SSE program, and the therapist verbally emphasized the need to maintain ABB throughout the movement. The CG was allowed to breathe normally without special emphasis on breathing.

The exercise intervention comprised a 10-min warm-up, 30-min of SSE, and 10-min cool-down. SSE programs were divided into an early phase (0–8 weeks), intermediate phase (8–16 weeks), and late phase (16–24 weeks) and included exercises to improve spinal stability and strength.^[27]^ The early phase was performed on stable ground, and the late phase performed SSE on the progressively unstable ground using a gym ball or balance pad. In this study, the OMNI Resistance Exercise Scale was used to set the exercise intensity (0, extremely easy; 10, extremely hard), and the exercise was performed on a scale between 5 and 7.^[28]^ If symptoms such as pain or numbness in the lumbar spine or lower extremities appeared during the exercise, the intensity was decreased. The SSE program used in this study is summarized in Table 1.

**Table 1:** Overview of the spine stabilization exercise program.

	Early phase (0–8 wk)	Intermediate phase (8–16 wk)	Late phase (16–24 wk)
SSE program	- Prone kneeling lumbar pelvic rhythm - Sitting pelvic tilt using a gym ball - Heel slid - Tabletop position - Leg lowering - Prone Lying gluteal brace - Side-Lying spine lengthening - Shoulder bridge - Four-point Kneeling leg movement - Sitting knee raise	- High kneeling hip hinge - Pelvic shift with leg lift - Bridge with leg left - Bird dog - Dead bugs - Crunch - Medicine twist in supine position - Front planks - Side-lyinghip lift - Wall squats	- High kneeling hip hinge - Pelvic shift with leg lift - Side-lying body lift (Side bridge) - Dead bugs on a foam roller - Bird dog with a resistance band - Crunch with a gym ball - Reverse bride with a gym ball - Basic superman with a gym ball - Medicine throwing and caching on a mobile surface

SSE = spine stabilization exercise.

### 2.3. Outcome measurement

In this study, the LLA was the primary outcome, and the lateral view was obtained through X-ray imaging (Accury-625R, Dk, Medical system, Seoul, Korea) that included the lumbar spine and pelvis, with the participant in a standing position. To visualize spine alignment, the participant was asked to raise both arms over their head during imaging. After imaging, the LLA was measured according to the Cobb angle measurement method using Picture Archiving and Communication System (PACS, Viewrex, HechEhim, Guro, Korea) by radiologists not involved in the study. The program measured the lordosis angle through the upper endplate of the first lumbar vertebra and lower endplate of the fifth lumbar vertebra using the Cobb angle measuring tool.^[29]^ Measurements were performed 3 times, and the average value was used when the Cronbach alpha value was ≥0.8.

The Medx extension machine (Medx 96, Ocala, FL) was used to measure the isometric spine extensor strength. The lumbar extensor strength was evaluated at 0°, 12°, 24°, 36°, 48°, 60°, and 72° in the range of spinal flexion. Before the start of the test, the measurement equipment and test method were fully explained to the participant, and a pelvic restraint was applied and fixed using pelvic and head restraints to limit the movement of the lumbar spine. During the measurement, verbal feedback was given to allow the participant to exert maximum muscle strength, and approximately 10 seconds of rest was given between each angle measurement. Each angle was measured twice, and the average value was used.^[30]^

Pain was assessed using a visual analog scale score range of 0 to 10 points, and lumbar function was assessed using the Oswestry disability index,^[31]^ which includes an evaluation of 10 categories, each comprising 6 items scored from 0 to 5 (score range, 0–50 points); however, this study excluded sex life, which was considered sensitive, and evaluated the remaining 9 items. All measurements were performed at baseline and after 12 and 24 weeks.

### 2.4. Statistical analysis

SPSS program version 23.0 (IBM Corp., Armonk, NY) was used for data analysis. Data normality was verified using the Kolmogorov–Smirnov test and analyzed using the parametric statistical method. An independent *t* test was used to analyze differences for continuous data, and Fisher exact test was used for nominal data of baseline characteristics. Differences in the LLA, spinal extensor strength, pain, and function from baseline to 24 weeks of follow-up were analyzed using a 2-way repeated measures analysis of variance (group × time). Post hoc analyses were performed to identify the progression within each group using the Bonferroni correction, and differences between groups were analyzed using an independent *t* test. Differences between the groups were expressed by interpreting the ES of Cohen *d* as small (<0.2), medium (0.5), and large (>0.8). A *P* value < .05 was considered statistically significant.

## 3. Results

In the ABBG, 2 participants dropped out, and 33 participants (10 male/23 female) were finally analyzed. In the CG, 1 participant dropped out, and 34 participants (10 male/24 female) were finally analyzed (Fig. 2). The mean age of participants in the ABBG was 44.8 ± 10.8 years, and the mean age of participants in the CG was 43.0 ± 10.6 years. The baseline characteristics of participants are shown in Table 2.

**Table 2:** Baseline characteristics of study participants (n = 67).

	ABBG (n = 33)*	CG (n = 34)*	*P* value
Age, yr	44.8 ± 10.8	43.0 ± 10.6	.504
Height, cm	166.4 ± 6.8	164.2 ± 8.6	.235
Weight, kg	66.1 ± 8.7	64.4 ± 9.3	.424
BMI, kg/m^2^	23.8 ± 1.7	23.8 ± 1.8	.991
Male, n (%)	10 (50%)	10 (50%)	.897
Female, n (%)	23 (48.9%)	24 (51.1%)	

ABBG = abdominal bracing group, BMI = body mass index, CG = Control group, n.s. = non-significant *P* value.

* Data are expressed as mean ± standard deviation.

### 3.1. Changes in lumbar lordosis angle

The LLA measurement from baseline to week 24 are shown in Table 3. In both the groups, the LLA was significantly increased over time, which was confirmed in the post hoc analysis (ABBG: baseline vs 12 weeks*, P* < .001 and 12 vs 24 weeks*, P* = .003; CG: baseline vs 12 weeks*, P* < .001 and 12 vs 24 weeks, *P* = .02). However, there was no significant difference between the groups (ES = 0.33; *P* = .12).

**Table 3:** Lumbar lordosis angle, visual analog scale, and Oswestry disability index at follow-up measurements (mean ± standard deviation) and change score at 24 wk.

	Baseline		12 wk		24 wk		Mean change within groups (baseline-24 wk)*		Mean difference between groups (change in score from baseline-24 wk)^a^	*P* value†
	ABBG (n = 33)	CG (n = 34)	ABBG (n = 33)	CG (n = 34)	ABBG (n = 33)	CG (n = 34)	ABBG (n = 33)	CG (n = 34)		
LLA	32.4 ± 4.9	32.8 ± 5.1	35.5 ± 4.9§	34.6 ± 3.9§	36.6 ± 4.3§	35.2 ± 4.1‡	3.8 (2.9; 4.6)§	2.8 (1.9; 3.6)§	1.0 (−0.2; 2.2)	.12
VAS	5.6 ± 1.1	5.4 ± 1.0	3.6 ± 1.5§	3.5 ± 1.1§	1.5 ± 1.0§	2.0 ± 0.8§	−4.2 (−4.6; −3.7)§	−3.4 (−3.8; −3.0)§	−0.8 (−1.4; −0.2)‡	.04
ODI	16.6 ± 3.5	17.1 ± 3.1	10.8 ± 3.5§	12.1 ± 3.4§	6.3 ± 2.1§	9.1 ± 3.3*§	−10.3 (−11.6; −9.0)*	−8.1 (−9.4; −6.7)§	−2.2 (−4.1; −0.4)‡	.02

ABBG = abdominal bracing group, CG = control group, LLA = lumbar lordosis angle, n.s. = non-significant *P* value, ODI = Oswestry disability index, VAS = visual analog scale.

* Reported as mean (95% confidence interval).

† Interaction effect by 2-way repeated measures analysis of variance.

‡ *P* < .05.

§ *P* < .001.

### 3.2. Spine extensor strength

**Figure 3.**
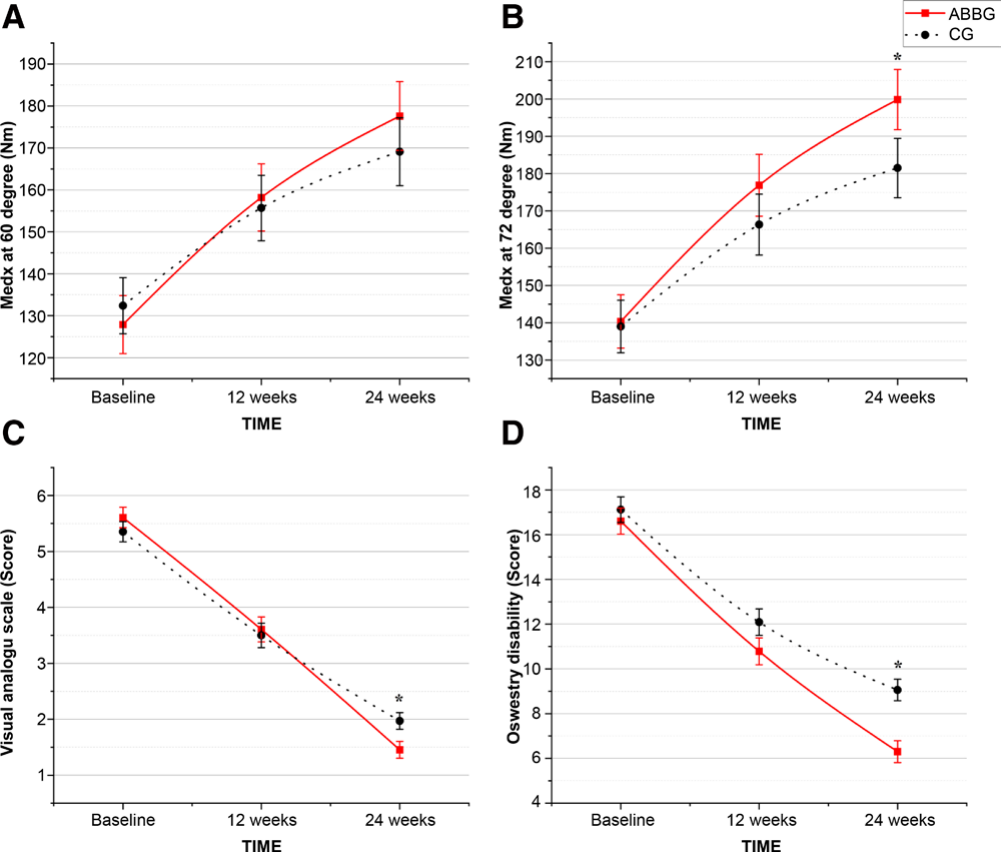
Changes in spine extensor strength, visual analog scale (VAS) score, and Oswestry disability index (ODI). Changes in spine extensor strength at (A) 60° and (B) 72°; (C) VAS score and (D) ODI from baseline to 24 wk. ^*^P < .05 for the independent *t* test (mean and standard error and 95% confidence interval).

The results of the LLA from baseline to weeks 24 are shown in Table 4. In both the groups, spine extensor strength at all degrees (0°, 12°, 24°, 36°, 48°, 60°, and 72°) was increased significantly (*P* < .001, respectively). In the post hoc analysis, the ABBG had an increased LLA over time (baseline vs 12 weeks, *P* < .001 and 12 vs 24 weeks, *P* = .006 at 0°; baseline vs 12 weeks, *P* < .001; and 12 vs 24 weeks, *P* < .001 at 12°, 24°, 36°, 48°, 60°, and 72°), and the same was true for the CG (baseline vs 12 weeks, *P* < .001; 12 vs 24 weeks, *P* < .001 at 0°, 24°, 36°, 48°, 60°, and 72°; baseline vs 12 weeks, *P* = .001; and 12 vs 24 weeks, *P* = .006 at 12°). There were no significant differences between the 2 groups at 0°, 24°, 36°, and 48°. However, the interaction effect between group and time was statistically significant at a spine flexion angle of 60° and 72° (ES = 0.18, *P* = .03 and ES = 0.39, *P* = .04, respectively). Post hoc analysis results between the groups are shown in Figure 3A–B.

**Table 4:** Changes and differences in spine extensor strength.

	Mean change*		Mean change†		Mean difference‡
	ABBG (n = 33)	CG (n = 34)	ABBG (n = 33)	CG (n = 34)	
0°	19.8 (13.0; 26.6)¶	25.5 (18.8; 32.2)¶	30.4 (22.2; 38.6)ǁ	41.1 (33.1; 49.2)¶	−10.8 (−22.3; 0.7)
12°	24.4 (17.5; 31.2)¶	26.1 (19.3; 32.9)¶	38.1 (29.6; 46.6)¶	36.5 (28.1; 44.9)ǁ	1.7 (−10.3; 13.6)
24°	27.1 (21.0; 33.1)¶	25.8 (19.8; 31.7)¶	38.3 (29.6; 47.1)¶	38.4 (29.8; 47.0)¶	−0.1 (−12.4; 12.1)
36°	27.5 (20.3; 34.7)¶	23.1 (16.1; 30.2)*¶	44.4 (34.8; 54.0)¶	38.1 (28.6; 47.5)¶	6.4 (−7.1; 19.8)
48°	30.6 (24.2; 37.0)¶	24.5 (18.2; 30.8)¶	46.4 (37.6; 55.2)¶	39.5 (30.8; 48.1)¶	6.9 (−5.4; 19.2)
60°	30.2 (24.3; 36.2)¶	23.3 (17.5; 29.2)¶	49.7 (40.8; 58.6)¶	36.7 (27.9; 45.5)¶	13.0§ (0.5; 25.5)
72°	36.5 (30.6; 42.5)¶	27.3 (21.5; 33.2)¶	59.5 (52.8; 66.2)¶	42.5 (35.9; 49.1)¶	17.0§ (7.6; 26.5)¶

ABBG = abdominal bracing group, CG = control group.

* Mean change within the group from baseline to 12 wk (95% confidence interval).

† Mean change within the group from baseline to 24 wk (95% confidence interval).

‡ Mean difference between groups for mean change from baseline to 24 wk (two-way repeated measures analysis of variance; 95% confidence interval).

§ *P* < .05.

ǁ *P* < .01.

¶ *P* < .001.

### 3.3. Pain and function

The pain and function results from baseline to week 24 are shown in Table 3. In both the groups, pain was decreased significantly over time. In the post hoc analysis, the pain in the ABBG improved over time (baseline vs 12 weeks and 12 vs 24 weeks, *P* < .001, both), as did that in the CG (baseline vs 12 weeks and 12 vs 24 weeks, *P* < .001, both). The interaction effect between group and time was statistically significant (ES = 0.55 and *P* = .04). Post hoc analysis results between the groups are shown in Figure 3C.

In both the groups, function (disability index) decreased over time. In the post hoc analysis, the function of the ABBG improved over time (baseline vs 12 weeks and 12 vs 24 weeks, *P* < .001, both), as did that of the CG (baseline vs 12 weeks and 12 vs 24 weeks, *P* < .001, both). The interaction effect between group and time was statistically significant (ES = 1.01, *P < *.02). Post hoc analysis results between the groups are shown in Figure 3D.

## 4. Discussion

According to the results of this pilot study, adding ABB to SSE for patients with nonspecific CLBP was more effective on lumbar extensor strength, pain, and function than SSE alone. However, we were unable to confirm any benefit that the addition of ABB could have on the LLA.

Although it is debated whether or not the LLA in the sagittal plane decreases or increases with age, a recent meta-analysis has reported that the LLA is significantly smaller in participants with LBP.^[32]^ Moreover, reduced LLA increases stress and load on the intervertebral disc, which in turn induces disc degeneration.^[33]^ Reduced LLA is not an absolute cause of LBP; however, it is one of the major risk factors that can predict LBP.^[34]^

To improve this reduced LLA, some researchers applied exercise intervention. In a study of 32 participants divided into an SSE group of 12 who used SSE for 12 sessions and a CG of 16 who used physical therapy alone, Hosseinfar et al reported that LLA recovery was confirmed in the SSE group.^[35]^ Woo et al also reported that the LLA increased after SSE 5 times a week for 30 minutes for 4 weeks among 15 participants.^[2]^ In our study, it was confirmed that the LLA increased in both the groups. We suggest that the increase in the LLA is associated with an improvement in the imbalance between the muscles related to pelvic tilt and those around the spine.^[34]^ Strong spinal extensors with weak spinal flexors cause the pelvis to tilt anteriorly, while strong flexors with weak spinal extensors cause the pelvis to tilt posteriorly.^[36,37]^ In our study, it was confirmed that the spine extensor strength increased in both the groups, and it is believed that this increased the anterior tilt of the pelvis, resulting in increased LLA in both the groups. However, the results of this study revealed that there was no significant difference in the LLA following the addition of ABB to SSE. We believe that this result could be due to the fact that although ABB increases IO activity,^[23]^ the IO does not directly affect the movement of the pelvis. Further studies on the activation of deep muscles, such as the IO and changes in pelvic tilt, will be needed in the future.

Spinal extensor strength also increased at all angles over time in both groups, but the interaction effect between group and time was confirmed at a spinal flexion angle of 60° and 72°. We provide evidence that these results are due to the activation of deep muscles, such as the IO and TrA, and an increase in IAP.^[23–25]^ The IO and TrA are considered important muscles that provide stability to the spine and increase intermuscular coordination during physical activity.^[38]^ Increased IAP is also known to contribute to spinal stabilization and increase trunk power and muscle strength.^[37,39]^ In an electromyography study by Maeo et al,^[23]^ even compared with dynamic exercise, including trunk flexion/extension exercise, ABB could lead to higher IO activation. Additionally, Moghadam et al reported that ABB activates the TrA more efficiently compared with abdominal drawing.^[25]^ Moreover, Tayashiki et al reported that ABB is an effective way to improve IAP.^[39]^ An additional study by Tayashiki et al also confirmed our data, where the authors reported that ABB increased isometric trunk extension by 14.4%, hip extension by 34.7%, and the maximum lifting force by 15.6%.^[40]^ Spinal extensor strength is important in patients with CLBP. Reduced spinal extensor strength and endurance, and fatigue are associated with LBP, and weakness of the spinal extensors is also associated with acute lumbar spinal injury.^[41]^ Moreover, a randomized controlled study by Steele et al,^[42]^ considered the role of lumbar extensors during walking, and the weakening of the extensor muscles in patients with CLBP, and showed that resistance exercise of the lumbar extensors reduces the variability of the gait pattern, especially in the sagittal plane.^[42]^ Therefore, we believe that the addition of ABB to SSE is effective in reducing symptoms and risk in patients with CLBP improving in spinal extensor strength.

Pain was improved over time in both the groups, and it was found that pain in the ABBG was better at 24 weeks. We believe that these results were demonstrated by deep muscle activation and increased IAP through ABB. Aleksiev et al previously performed a 10-year follow-up study designed as a randomized control trial and reported that spinal strength with ABB showed improvement in LBP compared with spinal strength without ABB, spinal flexibility, and spinal flexibility exercises with ABB.^[22]^ In their study, all the groups showed improvement in back pain up to 2 years after the start of the study, but the group that did not include ABB experienced increased pain again.^[22]^ This corroborated our finding that the application of a breathing method, such as ABB, improves LBP.

Finally, at 24 weeks after treatment, a significant difference was noted in the function, as evaluated by the Oswestry disability index, between the groups; it also showed the largest ES compared to other dependent variables. We believe that deep muscle activation, increased IAP, and concomitant contractions of the trunk muscles by ABB will contribute to stabilization of the spine and to improve pain and function in patients with LBP. Lee et al reported that the thickness of the IO, EB, and TrA increased, and the isometric strength of the lumbar extensor muscles improved as a result of applying straight leg raise with ABB.^[43]^ Additionally, Grenier et al reported that ABB improved lumbar stability by 32%, with a 15% increase in lumbar compression, which contributed more to spinal stability than the implementation of the abdominal hollowing technique.^[44]^ The increase in the stabilization of the lumbar spine induced by ABB facilitates a reduction in the stress and damage of the lumbar spine occurring in daily life, and functionally excellent results can be obtained because the performance of daily life is increased. According to a study by Vera-Garcia et al, compared to other breathing methods, ABB resulted in decreased displacement of the lumbar spine, along with co-contraction of the trunk muscles in response to a sudden trunk perturbation, thereby increasing spinal stability.^[45]^

Based on these findings, the LLA may be improved with SSE, although this does not appear to provide evidence that the LLA is a determinant factor for differences in spine extensor strength, pain, and function. Instead, it can be concluded that these results were caused by the activation of deep spinal muscles and an increase in IAP caused by the addition of ABB to SSE.

This study has several limitations. First, the strength of other muscle groups could not be evaluated, as we only measured the spine extension strength. Second, as the functional evaluation relied solely on a questionnaire and lacked physical assessment, making it challenging to evaluate the exact functional capabilities of patients. Finally, although we confirmed muscle strength in this study, we could not confirm morphological changes in the abdominal muscles due to the intervention, as measurements, such as muscle thickness evaluation, were not performed. In some studies, the TrA, IO, and external oblique muscles were examined using ultrasound during exercises such as ABB and abdominal hollowing in patients with CLBP, employing a reliable evaluation method^.[46,47]^ Therefore, future studies are warranted to evaluate abdominal muscles thickness and functions, such as balance ability, along with evaluation of other muscle groups.

In this pilot study, we were unable to confirm the reason behind the changes in the LLA and their effects on pain and function, although it was demonstrated that SSE results in the LLA changes in patients with CLBP.

Moreover, adding ABB had no additional effect on changes in the LLA, although it was confirmed that it was more effective in improving spine extensor strength, pain, and function than SSE alone. Therefore, in this study, we recommend adding ABB to SSE to maintain lordosis in the lumbar spine and to improve symptoms in patients with CLBP.

## Acknowledgments

We would like to thank Editage for helping with English proofreading.

## Author contributions

**Conceptualization:** Jae-Keun Oh.

**Data curation:** Si Won Park.

**Investigation:** Han Soo Park.

**Methodology:** Han Soo Park.

**Resources:** Han Soo Park.

**Supervision:** Jae-Keun Oh.

**Writing – original draft:** Han Soo Park.

**Writing – review & editing:** Si Won Park.
